# O.5.1-3 Co-creation of an active transportation promotion intervention: a concept mapping study

**DOI:** 10.1093/eurpub/ckad133.233

**Published:** 2023-09-11

**Authors:** Nuttanun Siriaporn, Audrey de Nazelle, Anne Vuillemin

**Affiliations:** Université Côte d'Azur, Nice, France; Imperial College London, London, UK; nuttanun. siriaporn@etu.univ-cotedazur.fr; Université Côte d'Azur, Nice, France

## Abstract

**Purpose:**

Active transportation (AT) is an efficient way to increase daily physical activity and reduce pollution from the transportation sector. Mhealth has contributed to behavior change in fields like physical activity and has been explored to encourage AT. Most interventions, however, did not incorporate evidence-based behavior change techniques and were not co-created with potential users. We aim to understand how AT can be promoted via mhealth intervention by using a bottom-up participatory approach.

**Methods:**

Concept mapping was done online via GroupWisdom platform, with the public of Alpes-Maritimes County, France. Participants were asked to brainstorm, sort, and rate ideas about mobile app features to encourage AT. Multidimensional scaling algorithm and hierarchical cluster analysis were used to create visual cluster maps to illustrate the participants’ conceptual thinking of the topic. We compared the data by participants’ age, education, and typical transportation mode to understand how different groups perceived each idea and clusters.

**Results:**

Participants generated 44 unique ideas that they believe would encourage AT behaviors. Six themes emerged, from highest to lowest cluster rating: Infrastructure (n = 9 ideas) and Itinerary (n = 9 ideas), which were consistently rated across groups; Contact with government (n = 4 ideas); Data (n = 12 ideas), with the most number of ideas generated; App usability (n = 6 ideas); and Legislation and code of conduct (n = 4 ideas), with the least number of ideas.

**Conclusions:**

Our findings showed various ways AT can be promoted. Even though the participants were asked about mobile apps features, they quickly pointed out ideas unrelated to mobile apps. The built environment and infrastructure are clearly the most important factors to promote AT, for our participants. However, they have also identified other soft measures like information, participation in decision making, and social aspects that can contribute to behavior change and support sustainable and healthy modes of transportation. Therefore, a systemic and multifaceted approach is recommended to promote AT.

**Support/Funding Source:**

This work was supported by the French government, managed by the Agence Nationale de la Recherche under the project Investissements d'Avenir UCAJEDI with the reference n° ANR-15-IDEX-01.

